# Adolescent THC exposure does not sensitize conditioned place preferences to subthreshold d-amphetamine in male and female rats

**DOI:** 10.12688/f1000research.14029.2

**Published:** 2018-09-27

**Authors:** Robin J Keeley, Cameron Bye, Jan Trow, Robert J McDonald

**Affiliations:** 1 University of Lethbridge, 4401 University Drive, Lethbridge, AB, T1K 3M4, Canada; 2 National Institute on Drug Abuse, 251 Bayview blvd, Suite 200, Baltimore, MD, 21224, USA

**Keywords:** THC, adolescence, d-amphetamine, strain, sex, conditioned place preference

## Abstract

The acute effects of marijuana consumption on brain physiology and behaviour are well documented, but the long-term effects of its chronic use are less well known. Chronic marijuana use during adolescence is of increased interest, given that the majority of individuals first use marijuana during this developmental stage , and  adolescent marijuana use is thought to increase the susceptibility to abusing other drugs when exposed later in life. It is possible that marijuana use during critical periods in adolescence could lead to increased sensitivity to other drugs of abuse later on. To test this, we chronically administered ∆ ^9^-tetrahydrocannabinol (THC) to male and female Long-Evans (LER) and Wistar (WR) rats directly after puberty onset. Rats matured to postnatal day 90 before being exposed to a conditioned place preference task (CPP). A subthreshold dose of d-amphetamine, found not to induce place preference in drug naïve rats, was used as the unconditioned stimulus. The effect of d-amphetamine on neural activity was inferred by quantifying *cfos* expression in the nucleus accumbens and dorsal hippocampus following CPP training. Chronic exposure to THC post-puberty had no potentiating effect on a subthreshold dose of d-amphetamine to induce CPP. No differences in *cfos* expression were observed. These results show that chronic exposure to THC during puberty did not increase sensitivity to a sub-threshold dose of d-amphetamine in adult LER and WR rats. This supports the concept that THC may not sensitize the response to all drugs of abuse.

## Introduction

Marijuana is one of the most commonly used drugs of abuse worldwide ^1^, and the psychoactive properties of marijuana are a result of the actions of ∆ ^9^-tetrahydrocannabinol (THC) ^2, 3^. Chronic marijuana use is associated with an increased risk of psychosis and depression ^4^, and these relationships are even more concerning when use occurs during adolescence (for example, 5– 7). Furthermore, there is the possibility that increased marijuana use in adolescence increases the likelihood of engaging with and becoming dependent on other substances, although this topic remains contentious ^8, 9^. In addition to the reported increased sensitivity of the adolescent period to the effects of marijuana, sex may also play a role in the consequences of both short- and long-term marijuana use with females more sensitive to depression and anxiety following marijuana exposure in adolescence ^10^.

In addition to sex differences in the outcome of adolescent marijuana use, genetic background, including rat strain, can change the long-term consequences of THC exposure ^11, 12^. Rat strains vary on measures related to learning and memory ^13– 20^, anxiety ^20^ and development ^12^ as well as in response to drugs of abuse ^21– 27^. Given that rat strains are used interchangeably in drug abuse research despite their innate differences, the inclusion of multiple strains of rat in any one study can help determine the strength and reproducibility of the long-term consequences of marijuana. 

Marijuana use during adolescence may increase the likelihood of engaging in other physiologically and sociologically harmful drugs of abuse in adulthood. THC administration can potentiate the response to opioids ^28^ and nicotine ^29^, through the facilitation of brain reward mechanisms ^30, 31^. However, the interaction between the consumption of one drug of abuse and initiating use of another is complex, and individual differences may predict sensitivity to other drugs, including amphetamine ^21, 25, 32– 34^. The use of multiple rat strains, including Long-Evans (LER) and Wistar (WR) rats that have previously been observed to have differential sensitivity to THC, can model individual differences in response to THC.

This study sought to determine the long-term consequences of THC administration during the post-pubertal period in two previously studied strains of rats ^11^. Following systemic administration of THC for 14 days after puberty onset, rats were aged to 90 days, at which point all rats were trained in a conditioned place preference (CPP) task to a subthreshold dose of d-amphetamine. It was hypothesized that if a particular strain and sex group was more sensitive to the effects of THC and if THC exposure increased the sensitivity to other drugs of abuse, sensitive rats would develop CPP to the sub-threshold dose of d-amphetamine and show increased neural activation, as inferred by protein expression of the immediate early gene, *Cfos*, in reward (nucleus accumbens) and context-specific (dorsal hippocampus) brain regions. However, if THC administration does not increase the sensitivity of rats to amphetamine, then no strain or sex group should show CPP behaviour in response to a subthreshold dose of d-amphetamine and no differences in *Cfos* expression should be observed.

## Methods

An experimental timeline of all procedures can be found in Supplementary Figure 1.

### Experiment 1: Determining a subthreshold dose of d-amphetamine in drug naïve rats

***Subjects.*** Subjects were purchased and shipped from Charles River (Semmeville, Quebec) as adults (250–300g) (LER female: N = 16; LER male: N = 24; WR female: N = 16; WR male: N = 16). All rats were housed in standard laboratory conditions (21°C and 35% relative humidity; 12D:12L) in Plexiglas tubs (46cm × 25cm × 20cm) with *ad libitum* access to food and water. All rat handling and procedures were done in accordance to the University of Lethbridge’s Animal Welfare Committee and the Canadian Council on Animal Care guidelines.

**Figure 1.**
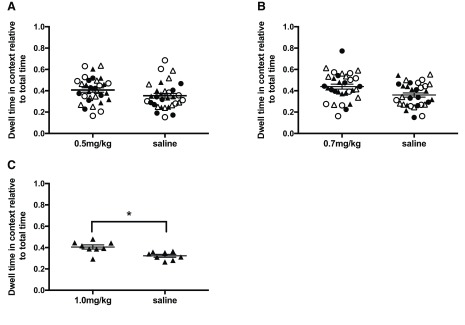
Dwell time in the previously paired (black) and unpaired (white) contexts during CPP. **A**. 0.5mg/kg d-amphetamine. **B**. 0.7mg/kg d-amphetamine. **C**. 1.0mg/kg d-amphetamine. Note 0.5 and 0.7mg/kg d-amphetamine was tested in all strain and sex groups and 1mg/kg was tested only in LER males to confirm previously published work. * p < 0.05. Individual data plus mean and SEM. LER females (closed circle), LER male (closed triangle), WR female (open circle), WR male (open triangle).

***D-amphetamine doses***. Drug naive adult rats were tested using three doses of d-amphetamine, 0.5mg/kg, 0.7mg/kg and 1mg/kg (0.49mg/ml d-amphetamine in saline, Sigma Aldrich). These doses were chosen as 1mg/kg of d-amphetamine has been shown to induce CPP in multiple research groups (as reviewed in 35) and was confirmed here in naïve LER male rats ( Figure 1C). N = 8 for each strain, sex and drug dosage group.

***CPP: Apparatus and training.*** *Apparatus* – A similar apparatus and procedure to that used for discriminative appetitive ^36, 37^ and fear conditioning ^38, 39^ to context tasks were implemented here. Briefly, opaque Plexiglas contexts that differed in shape (triangle versus square), colour (black versus white) and odour (amyl acetate versus eucalyptus), were connected with a grey alleyway. Both contexts and the alleyway were placed upon a clear Plexiglas table, and underneath the table, a mirror was inclined at a 45° angle which allowed for viewing by both an observer and a video camera.

*Training* – Pre-exposure: Rats were placed in the grey alleyway and allowed to freely explore both contexts for 10min then returned to their home cage. Dwell time in each chamber was recorded by an observer.

Training: The context to be paired with d-amphetamine injection (paired) and the context to be paired with a saline injection (unpaired) were assigned to each rat in a counterbalanced, quasi-random fashion. For training, rats were given 6 consecutive daily exposures ^35^, where they were given an injection of either saline or d-amphetamine then placed in one of the contexts for 30min. Injection type and context exposure alternated each day.

Preference: Rats explored the contexts connected by a grey alleyway for 10min. Dwell time in both contexts was recorded. All observers were blind to conditions.

### Experiment 2: CPP in adolescent THC exposed rats

***Subjects, puberty onset and drug administration***. Subjects the offspring of rats purchased from Charles River; they were acquired, bred and handled as previously described ^11, 12, 17^. Briefly, male and female LER and WR (N = 9/strain and sex group) were obtained from Charles River (Semmeville, Quebec) and were acclimated for 2 weeks before breeding. Pups were weaned at postnatal day 21 (p21) and placed into sex-matched pairs or triplets. N = 8 for all strain and sex groups for all experiments.

Puberty onset, group assignment and injection procedures were conducted as previously described ^12^. Puberty onset was determined using the external features of the genitalia (vaginal opening and preputial separation), which correlate with gonadal hormone changes associated with puberty ^40, 41^. On weaning day, rats were assigned to their experimental groups: handled control (CON), vehicle (VEH; 1:1:18 ethanol:cremaphor:saline) or 5mg/kg THC (THC). I.p. injection procedures and handling were conducted as previously described ^11^. On the day of determination of puberty onset, rats were brought to a dark injection room. All rats were weighed before treatment. All rats received treatment for 14 consecutive days following determination of puberty onset. After the treatment period, rats were aged to adulthood (p90) before behavioural testing.

***CPP to a subthreshold Dose of d-amphetamine: Apparatus & training***. From the results of Experiment 1 (see Results section), a subthreshold dose of d-amphetamine was determined to be 0.7mg/kg. This dose was used for all rats exposed to adolescent THC. Apparatus and training were conducted as described.

***Perfusion & fixation***. *Cfos* protein is present in neurons that were active 20–30min after an experience ^42^, and in rats, d-amphetamine will reach the brain within 5min of an i.p. injection and remain stable for 1hr ^43^. Any *cfos* protein signal detected 1hr after d-amphetamine injection represents the neurons active 30min after d-amphetamine injection. One week after the final day of CPP, rats were injected with a single 1mg/kg dose of d-amphetamine and sat in their home cage for 1hr. Rats were euthanized with a single i.p. injection of sodium pentobarbital (120mg/kg) and transcardially perfused with approximately 150mL of 1x phosphate-buffered saline (PBS) followed by 4% paraformaldehyde (PFA) in 1xPBS. Brains were immersion fixed in 4% PFA in 1xPBS. PFA was replaced 24h after perfusion with 30% sucrose and 0.2% Na azide in 1xPBS. Brains were sectioned at 40µm using a cryostat (CM1900, Leica, Germany) and placed directly into Eppendorf tubes containing 0.2% Na azide in 1xPBS.

***Cfos immunohistochemistry & quantification***. The amount of cfos protein was stained as previously described ^44^. Briefly, free-floating tissue was washed (1xPBS), followed by a 30min quenching step (0.3% H _2_O _2_ in 1xPBS). Tissue was blocked (1.5% goat serum in 0.3% triton-X 1xPBS) for 30min then incubated in 1° antibody (rabbit; 1:1000, 0.33% triton-X in 1xPBS with 1.5% normal goat serum; Santa Cruz, California) for 24hrs. Then, tissue was washed followed by a 24hr incubation in 2° antibody (anti-rabbit; 1:1000, Vector Labs, Canada) at room temperature. On the third day, tissue was washed then placed in AB Complex (Vector labs, Canada) for 45min. Tissue was washed then bathed for 5min in a 0.5% 3,3’-diaminobenzidine (DAB) solution (1xPBS with NiCl _2_-6H _2_O and 0.05% H _2_O _2_). Sections were washed then mounted on 1% gelatin coated slides left to dry for 24hrs, dehydrated and coverslipped with Permount.

Representative images from NAc and dorsal hippocampus were taken and quantified using particle analysis in Image J (NIH, USA). Regions of interest were defined using the Rat Brain Atlas ^45^, and particles were counted per unit area.

### Vaginal cytology and determination of estrous cycle

Vaginal cytology and the determination of estrous cycle was conducted as previously described ^11, 12, 36^. Sterile Q-tips were dipped in sterile distilled water to collect samples onto standard glass slides (Vector labs, Canada). Vaginal smears were collected during all behavioural testing days and examined using brightfield microscopy on a Zeiss Axio Imager MT (Carl Zeiss, MicroImaging GmBH, Germany) using the 20X objective.

### Statistical analysis

All raw data can be found in the raw dataset. Statistical tests were conducted using SPSS (IBM, ver 17), and estrous cycle phase was used as a covariate. For Experiment 1, a repeated measures ANOVA was conducted for percent dwell time in either context with strain and sex as the between subjects factors. Since we were interested in whether a preference for one context over another had occurred, *a priori* ANOVA tests were conducted within each strain and sex group comparing dwell time in each context. We report partial η ^2^ for effect size and observed power for all results. We did not compare dwell times within animal pre- and post-training.

For Experiment 2, percent dwell time in the paired and unpaired contexts on the pre-exposure and preference days were compared within strain and sex groups using drug condition (group) as a between subjects factor. *A priori* hypotheses were established such that within each drug group and within each strain and sex group, comparisons between the paired and unpaired contexts were always conducted. For *cfos* quantification, between subjects comparisons within strain and sex groups were conducted in order to determine the effects of drug exposure on a specific strain and sex group. 

## Results

Estrous cycle did not significantly alter any of the results and was not included as a covariate in subsequent analyses.

### Experiment 1: Determination of a subthreshold dose of d-amphetamine

No initial preference nor any preference after training was observed for 0.5 ( Figure 1A) or 0.7mg/kg ( Figure 1B) of d-amphetamine for any strain or sex group (see Table 1 for statistical results). A dose of 1mg/kg d-amphetamine was used to confirm previous experiments and did induce significant place preference (see Figure 1C), thus 0.7mg/kg dose was considered subthreshold for all subsequent experiments.

**Table 1.** Statistical results for Experiment 1.

Dose of d-amphetamine	Effect	F	df	p	Partial η ^2^	Observed power (p < 0.05)
0.5mg/kg	Context	0.851	1, 31	0.183	0.056	0.261
0.7mg/kg	Context	3.854	1, 31	0.059	0.111	0.477
1.0mg/kg	Context	7.083	1, 7	0.032	0.503	0.629

### Experiment 2: CPP to a sub-threshold dose of d-amphetamine

**Figure 2.**
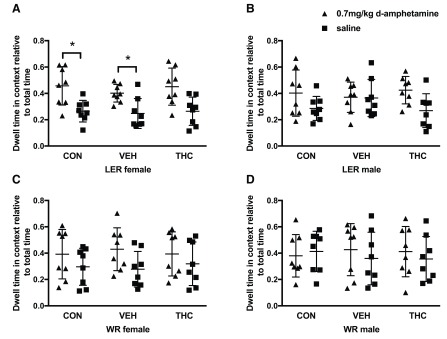
CPP to 0.7mg/kg d-amphetamine in THC exposed adolescent LER and WR male and female rats. Preference for **A**) LER females, **B**) LER males, **C**) WR females and **D**) WR males. * p<0.05.

There were no pre-existing bias to spend more time in the paired or unpaired context, regardless of strain, sex or drug administration. No interaction between drug or context were observed in any strain and sex group. On the preference day, LER females overall spent significantly more time in the paired context (F _(1, 21)_ = 17.483, p < 0.001; Figure 2A). No overall effect of group was observed. Individual comparisons within groups revealed that CON (p = 0.04) and VEH (p = 0.028) LER females spent significantly more time in the context paired with d-amphetamine. No such difference was observed within LER females exposed to THC, although this value did approach statistical significance (p = 0.065). LER males ( Figure 2B), WR females ( Figure 2C) and WR males ( Figure 2D) showed no significant effect of drug as well as did not show an overall preference for one context over the others (see Table 2 for statistical results).

**Table 2.** Statistical results for Experiment 2 preference.

Group	Effect	F	df	p	Partial η ^2^	Observed power (p < 0.05)
LER females	Group	1.688	2, 21	0.209	0.138	0.315
	Context	17.483	1, 21	< 0.001	0.454	0.978
	Group x Context	0.080	2, 21	0.923	0.008	0.061
LER males	Group	0.828	2, 21	0.451	0.073	0.173
	Context	3.432	1, 21	0.078	0.140	0.424
	Group x Context	0.809	2, 21	0.459	0.072	0.169
WR females	Group	0.277	2, 21	0.761	0.026	0.088
	Context	2.811	1, 21	0.108	0.118	0.360
	Group x Context	0.130	2, 21	0.879	0.012	0.067
WR males	Group	0.331	2, 21	0.722	0.031	0.096
	Context	0.169	1, 21	0.685	0.008	0.068
	Group x Context	0.194	2, 21	0.825	0.018	0.076

### *Cfos* immunohistochemistry

**Figure 3.**
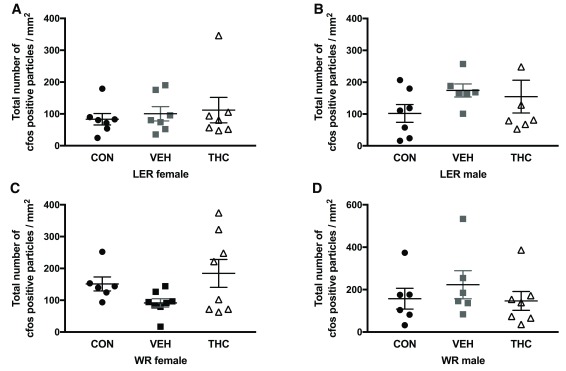
*Cfos* staining in dorsal hippocampus in LER and WR male and female rats exposed to THC as adolescents.

**Figure 4.**
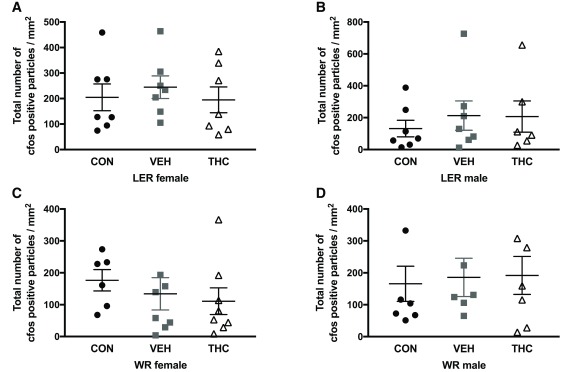
*Cfos* staining in NAc in LER and WR male and female rats exposed to THC as adolescents.

No significant effects were observed for any strain and sex group for *cfos* expression in dorsal hippocampus ( Figure 3) and NAc ( Figure 4) following a 1mg/kg injection of d-amphetamine. 

Raw data associated with Figure 1 – Figure 4 Click here for additional data file. Copyright: © 2018 Keeley RJ et al. 2018 Data associated with the article are available under the terms of the Creative Commons Zero "No rights reserved" data waiver (CC0 1.0 Public domain dedication).

## Discussion

Here, we report no long-term consequences of adolescent THC exposure on sensitivity to d-amphetamine in adulthood. We did observe that rearing environment affected sensitivity to d-amphetamine in LER females; CON LER females bred in house expressed CPP behaviour to a 0.7mg/kg dose of d-amphetamine, whereas those obtained from a commercial breeder (Charles River) did not. Additionally, using immediate early gene protein expression, we observed no significant effect of THC exposure following puberty onset in nucleus accumbens and dorsal hippocampus d-amphetamine-induced activation.

### No effect of THC

Adolescent THC exposure did not potentiate the adult response to d-amphetamine. D-amphetamine increases dopaminergic tone when systemically administered ^46– 49^ and is highly rewarding ^50, 51^. Given the premise that THC acts as a gateway drug, we assumed adolescent exposure to THC would potentiate reward circuitry, enhancing sensitivity to d-amphetamine.

Priming of amphetamine response by cannabinoids has been observed by some researchers ^52– 54^ and not others ^55^. Differences between among studies look for this effect include the dose, duration and starting age of exposure to THC as well as the timing of exposure to amphetamine, which one study reported amphetamine-primed reward to be dependent on the time since exposure to THC ^53^. However, our results should help mitigate many of these issues, as our dose of THC was relatively moderate, was given following the onset of puberty, which can be influenced by THC ^56^ and lasted throughout the adolescent period and in to early adulthood, all of which are reasonable analogues, given experimental constraints, to the human adolescent marijuana consumption experience. One possible explanation for the pattern of results obtained in the present study may be the use of CPP versus the self-administration paradigm. CPP is a standard metric for determining the rewarding properties of drugs of abuse and has been observed for multiple doses of drugs, including amphetamine ^35, 57– 59^. Future experiments should consider allowing animals to self-administer either THC or amphetamines, potentially looking at the correlations between self-administration of both drugs. Unfortunately, THC has proven problematic in self-administration paradigms in 57, 60, 61.

Previous studies have demonstrated priming effects of THC to other drugs of abuse. Increased self-administration of heroin or other opiates has been observed ^54, 62– 64^, partially dependent on cannabinoid receptors ^65^. Thus, the endogenous opioid system is particularly sensitive to the long-term consequences of THC. Indeed, given the increased abuse of prescription opiates, research examining the interplay between the endogenous cannabinoid and opioid systems could potentially prevent the transition of using marijuana to opiates. This research field has been of increasing interest; for example, the interplay between cannabidiol and morphine has been examined in mice ^66^. Thus, it remains possible that priming of THC is specific to drugs targeting the opioid system.

There is a potential link between cannabis use and schizophrenia ^67^, but a stronger link exists between amphetamines and psychosis ^68^. However, the existing evidence suggests that the link between schizophrenia and cannabis use is particularly strong amongst individuals with a genetic predisposition for schizophrenia ^67^, and the rodent models implemented here do not represent animal models of schizophrenia. Future research examining long-term changes due to THC exposure in adolescence in an animal model of schizophrenia may be better suited to address this research question.

### Effect of rearing environment

LER females bred in house at the University of Lethbridge expressed CPP to a 0.7mg/kg dose of d-amphetamine whereas those purchased from Charles River did not. Although this was not a main research question from this study, this effect was observed here, specifically and exclusively in LER females. Strain differences in response to amphetamine have been observed previously ^21, 22, 24, 25^. This strain and sex specific effect in response to amphetamine may be the result of the interplay between the stress system and monoaminergic function, which has been posited to explain differences in response to amphetamine between two other strains of rats, Fisher 344 and Lewis ^69, 70^. It is possible that differences in these systems may occur in LER females reared under different conditions ^71, 72^. Regardless, further understanding of this fascinating effect of strain and rearing conditions should be explored as it is clear that genetic and differences in rearing correlate highly with drug abuse in adulthood ^23, 34, 73– 75^.

Strain-dependent sex difference in response to amphetamines has been observed previously ^69^, although this effect was not observed in LER. Differential responses in one sex and not the other across strains are not uncommon (for example, 76), however most studies examining strain differences in response to drugs of abuse typically only use males (as discussed in 69). There is a tendency for females to be more sensitive to drugs of abuse, including amphetamine ^32, 69, 77– 80^, which is partially mediated through the endogenous hormonal rhythms of females ^81– 83^. Here, training days covered the extent of at least one full estrous cycle, and there was no significant effect of estrous cycle phase on CPP behaviour. However, it is possible that there were no significant effects of estrous cycle phase due to the sample size. Thus, we have identified that LER females are sensitive to rearing environment in relation to CPP behaviour in response to amphetamine. This kind of effect should not be underestimated as the implications of ignoring sex, strain, rearing differences and their interactions in research are being increasingly recognized by granting agencies and scientific organizations to contribute to individual differences and reproducibility in current neuroscience research.

## Conclusions

This study does not support a link between adolescent THC exposure and sensitivity to another drug of abuse, specifically d-amphetamine, where rats were tested for changes in sensitivity to d-amphetamine following long-term exposure of THC during adolescence. This is surprising, given the vulnerability of LER females to developmental perturbations (in this case, rearing environment) on d-amphetamine CPP. WR displayed stable behavioural profiles; neither rearing environment nor THC administration altered their response to a sub-threshold dose of d-amphetamine. Our previous research identified WR as resilient to the effects of adolescent THC exposure ^11^. Further research into discovering the mechanisms behind resiliency in these groups may help identify mechanisms that can be protective for groups at-risk to the development of addiction.

## Data availability

The data referenced by this article are under copyright with the following copyright statement: Copyright: © 2018 Keeley RJ et al.

Data associated with the article are available under the terms of the Creative Commons Zero "No rights reserved" data waiver (CC0 1.0 Public domain dedication). 

Dataset 1: Raw data associated with Figure 1– Figure 4. 10.5256/f1000research.14029.d196720 ^84^

## Supplementary material

Supplementary Figure 1

An experimental timeline of all procedures.
