# Supplementary figures and images for: Adolescent THC exposure does not sensitize conditioned place preferences to subthreshold d-amphetamine in male and female rats
Source: F1000Res. 2018 Sep 27;7:342. Originally published 2018 Mar 20. [Version 2] doi: 10.12688/f1000research.14029.2 (PMC5920568; doi:10.12688/f1000research.14029.2)

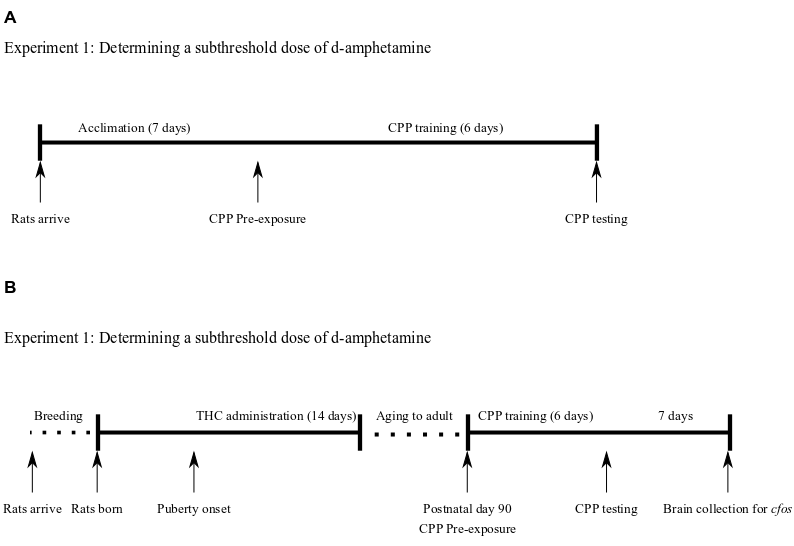

Supplement: Supplementary file 2 [file f1000research-7-17791-s0001.tgz › 8d1f7853-8b5a-4698-9f15-51a0d5b87864_SuppleFig1.png]
