# Supplementary material for: Adolescent THC exposure does not sensitize conditioned place preferences to subthreshold d-amphetamine in male and female rats
Source: F1000Res. 2018 Sep 27;7:342. Originally published 2018 Mar 20. [Version 2] doi: 10.12688/f1000research.14029.2 (PMC5920568; doi:10.12688/f1000research.14029.2)
Supplement: Peer review history

# Peer review: Referee response for version 1

**DOI:** 10.5256/f1000research.15248.r32202

**Published:** 2018-04-25

**Authors:** Jibran Y. Khokhar

**Affiliations:** 1 University of Guelph, Guelph, ON, Canada

This report by Keeley et al., examines the effects of adolescent THC exposure on conditioned place preference for d-amphetamine. While no effects of adolescent THC exposure were found on the CPP or the neural correlates (c-fos expression), the authors' inclusion of important factors such as strain and sex make this an important study to consider for those interested in studying the effects of developmental cannabinoid exposure on future risk for substance use. Suggested changes would help to improve the impact of this paper.

 Abstract: 

- Last Sentence: include "sub-threshold dose of" before d-amphetamine

Introduction:

- The introduction begins with mentions of psychosis, anxiety and depression, whereas the central questions being asked in this study are not related to those topics. The authors should think about setting the research question in the opening paragraph. 

Methods:

- Subjects: This section is unclear. Are the subjects being referred to here the parents of the rats used in the study?
- D-Amphetamine Doses: 1 mg/kg not listed here
- Statistical Analyses: Unclear whether dwell times were also compared within-animal pre- and post-training?

Results:

- Tables missing, but mentioned in the text. 

Discussion:

- Since the authors chose to discuss psychosis in the introduction, maybe they can discuss it in the context of the null results of d-amphetamine on c-fos expression, since an accentuated response to amphetamine is something that is seen in patients with schizophrenia.

An excellent review by Chadwick and Hurd ^1^ might help to formulate some of these thoughts. 

 Rearing environment was not one of the research objectives/hypotheses and the relevance to this discussion needs to be strengthened.

I have read this submission. I believe that I have an appropriate level of expertise to confirm that it is of an acceptable scientific standard.

## References

[1] ChadwickBMillerMLHurdYL: Cannabis Use during Adolescent Development: Susceptibility to Psychiatric Illness. *Front Psychiatry*.2013;4: 10.3389/fpsyt.2013.00129 129 10.3389/fpsyt.2013.00129 24133461PMC3796318

# Response

**Published:** 2018-09-10

**Authors:** Robin Keeley

**Affiliations:** National Institute on Drug Abuse, USA

We would like to thank the Referee for their thoughtful comments on our research. We have specifically addressed all comments that you mentioned and further clarified any statements as required. Specifically, we found your insight on the topic of schizophrenia particularly interesting. We believe that if this research study had been conducted in an animal model of schizophrenia, the results may have been significantly different.

Thank you again.

Best,

Robin J. Keeley

# Peer review: Referee response for version 1

**DOI:** 10.5256/f1000research.15248.r32404

**Published:** 2018-04-04

**Authors:** Ryan J. McLaughlin, Timothy G. Freels

**Affiliations:** 1 Department of Integrative Physiology & Neuroscience, Translational Addiction Research Center, Washington State University, Pullman, WA, USA; 2 Department of Psychology, University of Memphis, Memphis, TN, USA

This article contributes valuable insight into issues that are commonly observed in literature regarding the impact of adolescent Δ ^9^-tetrahydrocannabinol (THC) exposure on future drug-seeking behavior. Particularly, careful attention is directed towards the influence of *sex* (including estrous cycle) and *animal strain* on modulation of drug-seeking behavior by adolescent THC administration. These crucial factors likely contribute to conflicting findings reported on the influence of exogenous and endogenous cannabinoids on future drug consumption, and Keeley and colleagues illustrate the necessity of implementing sex and strain variables in experimental designs when addressing such research questions. As such, the authors’ findings provide a more informative story that will be useful in guiding legislation on marijuana use policies. The introduction of this article provides clear explication of hypotheses and expected results as well as suitable background information that warrants investigation of the authors’ research questions. However, there are some minor issues regarding the presentation of methods and statistical results. Additionally, there are some aspects of the discussion that could benefit from citation of supporting literature.

**Methods**

 In the statistical analysis section, the authors indicate that effect size and statistical power would be reported for all results (pg. 5), yet these data are not provided when presenting results in text. The analytical approaches utilized by the researchers are appropriate for the data; although, brief explanations of post-hoc statistical tests should be included.

 It is stated in the *Subjects *section (pg. 3), that all rats were purchased and shipped from Charles River. However, in the first paragraph of the discussion, the authors allude to an effect of rearing environment on d-amphetamine sensitivity. Specifically, Long Evans females bred in-house showed CPP to the 0.7 mg/kg dose, but this was absent in Long Evans females obtained from a commercial breeder. These data are valuable and should be included, but the analyses do not appear anywhere in the manuscript.

 Inclusion of an experimental timeline would increase the clarity of the researchers’ methodological approach. It would also provide a useful model for future researchers interested in testing the effects of THC administration on the reinforcing effects of drugs of abuse.

 Given that an observer scored dwell time in the CPP chambers, it would be beneficial to include the familiarity of the observer with animals’ drug treatment group. In other words, were the observers blinded to treatment conditions?

 The authors state that 3 doses of D-amphetamine were used, but only 2 doses are listed (0.5 and 0.7 mg/kg). We assume that the third dose is the vehicle (0 mg/kg), but that should be explicitly listed in the Methods (see pg. 3).

   

**Results**

 The results are clearly and concisely stated, and the provided figures summarize the data well. However, Table 1 referenced in the “Experiment 1: Determination of a subthreshold dose of d-amphetamine” section and Table 2 referenced in the “Experiment 2: CPP to a sub-threshold dose of amphetamine” section are not provided in the article (see pg. 5).

 In the caption for Figure 1, there is a space inserted into the word “Note”. Also in this caption it is stated that mean and “SEE” are provided. Presumably this was supposed to read SEM.

 The authors state that estrous cycle did not have any effect on the results (pg. 5). However, with only an N=8, it may simply be that there was not sufficient power to detect any differences. This should be briefly mentioned as a potential caveat.

**Discussion**

 On pg. 8, the authors indicate that “this study does not support a link between adolescent THC exposure and sensitivity to other drugs of abuse…”. Given that only sensitivity to d-amphetamine was assessed, it may be more reasonable to conclude from the provided results that adolescent THC exposure does not enhance sensitivity to d-amphetamine specifically.

 On pg. 7 – 8, the authors suggest that “…given the increased abuse of prescription opiates, research examining the interplay between the endogenous cannabinoid and opioid systems could potentially prevent the transition of using marijuana to opiates.”  This is an exciting prospect for future research, and some progress has been made in this regard. The authors may find it useful to include conclusions from Markos et al., (2017) ^1^ in which cannabidiol is shown to attenuate morphine CPP in mice.

We have read this submission. We believe that we have an appropriate level of expertise to confirm that it is of an acceptable scientific standard.

## References

[1] MarkosJRHarrisHMGulWElSohlyMASufkaKJ: Effects of Cannabidiol on Morphine Conditioned Place Preference in Mice. *Planta Med*.2018;84(4) : 10.1055/s-0043-117838 221-224 10.1055/s-0043-117838 28793355

# Response

**Published:** 2018-09-10

**Authors:** Robin Keeley

**Affiliations:** National Institute on Drug Abuse, USA

We thank the Referees for their thought-provoking as well as useful suggestions. We have addressed all the comments raised through modifications to the manuscript. Specifically, as in response to Dr. Khokar, we have clarified where the subjects were received from. Specifically, rats purchased from Charles River were used to determine the sub-threshold dose of d-amphetamine, and the offspring of rats purchased from Charles River and bred at the University of Lethbridge were used for the experiments examining the effects of adolescent THC to the response to amphetamine. To further clarify, we have included a supplementary figure as a timeline.
